# Effect of Male Involvement on the Nutritional Status of Children Less Than 5 Years: A Cross Sectional Study in a Rural Southwestern District of Uganda

**DOI:** 10.1155/2017/3427087

**Published:** 2017-12-04

**Authors:** Noel Kansiime, Daniel Atwine, Simpson Nuwamanya, Fred Bagenda

**Affiliations:** ^1^Department of Community Health, Mbarara University of Science and Technology, Mbarara, Uganda; ^2^Epicentre Mbarara Research Center-Uganda, P.O. Box 1956, Mbarara, Uganda

## Abstract

**Background:**

Undernutrition among children less than 5 years is still a public health concern in most developing countries. Fathers play a critical role in providing support in improving maternal and child health. There is little studied on male involvement and its measurement in child nutrition; therefore, this paper explores the level of male involvement in child feeding and its association with the nutritional status of the children less than 5 years of age.

**Methods:**

A cross sectional study among 346 households, 3 focus group discussions, and 4 key informant interviews were conducted in one rural district in Uganda. Adjusted odds ratios (aORs) and 95% confidence intervals (95% CI) of associated factors were estimated and focus group discussions and in-depth interviews were conducted and summarized into themes.

**Results:**

The study revealed the highest percentage of the males provided money to buy food for the children (93.6%), and only 9.8% have ever accompanied mothers to young child clinics.

**Conclusion:**

In this study, most males were involved in buying food for their children, and providing money for transport to young child clinics was associated with normal nutritional status of children less than 5 years in the study area.

## 1. Background

Undernutrition among children under 5 years of age is still a public health concern globally. It is estimated that 165 million were stunted, 101 million were underweight, and 52 million were wasted, in 2011, with high prevalence levels of undernutrition in Africa (36%) and Asia (27%) [1].

In Uganda, 33% are stunted, 14% are underweight, and 5% are wasted with notably high prevalence in the Southwestern (commonly known as a food basket of Uganda) and Karamoja regions of Uganda [2].

Under-five malnutrition directly and indirectly contributes to up to 60% of child mortality and projections suggested that over 520,000 children would die in Uganda as a consequence of underweight alone between 2006 and 2015 if the status quo was maintained [3]. Childhood stunting is an indicator of chronic malnutrition and is associated with compromised cognitive development and educational performance [4, 5]. The factors that contribute to under-5 malnutrition include large household sizes, unsteady income flow, age of introducing supplementary foods, lack of information on child care, and poor sanitation [6] and the immediate causes in developing nations continue to be the high disease burden such as from fever (40%), diarrhoeal disease (23%), and acute respiratory infections (15%), as well as inadequate dietary intake resulting from suboptimal infant feeding practices [3, 7, 8].

There has been recognition that, to improve maternal, infant, and young child nutrition, health structures need to attend to and support fathers, because they play a critical role in providing instrumental and emotional support to mothers and children [9]. Interventions that involve men as agents of positive change are relatively few in number, although research has indicated that men themselves, as well as their partners, would prefer that they play a more active role although the societal and health system norms often do not support this [10]. Engaging fathers is also important because of the significance of both the father-infant relationship (provision of physical and psychosocial support for mothers during the weaning period) and the couple relationship to overall individual and family well-being [10].

Fathers' involvement in parenting is associated with positive cognitive, developmental, and sociobehavioral child outcomes such as improved weight gain in preterm infants, improved breastfeeding rates, higher receptive language skills, and higher academic achievement [11]. Joint decision-making between women and their spouses also significantly increases women's final decision in uptake of other maternal health services such as Skilled Birth Attendance which also improves the newborns nutrition [12].

The Ministry Of Health of Uganda through its infant and young child feeding (IYCF) policy guidelines highlights the role of fathers in child feeding as participating in decision-making on infant and young child feeding, providing physical, psychological, and financial support during lactation, and developing an interest in promoting, supporting, and protecting infant and young child feeding for optimal feeding practices [13].

Male involvement in infant and young child feeding has been scarcely studied in Uganda yet evidence shows that men are key influencers of infant and young child feeding practices in most rural households [14]. The aim of this study was to determine the level of male partner involvement in infant and young child feeding and establish the relationship between level of male involvement and the nutritional status, of children under 5 years in a rural district of Southwestern Uganda.

## 2. Materials and Methods

A community based cross sectional study was conducted in 2013 among 346 households selected by multistage simple random sampling technique. Two subcounties were then randomly selected, from which two parishes were also randomly selected. At parish level, twenty-two villages were randomly selected and, by probability proportional to size sampling, a representative number of randomly selected households were recruited for this study. The households were the simplest sampling units for this study. In each household one male partner/caretaker and child aged 6–59 months pair were the study subjects. Three focus group discussions with groups of 8–12, males only, females only, and males with females, were separately conducted. Four key informant interviews were conducted with the parish councillor Kyeibanga, a nursing officer of Kitagata Hospital, and mother and father of previously malnourished under-five child. Qualitative methods were used to assess understanding of activities considered as male involvement in child nutrition and whether males participate in infant and young child feeding. Participants for the FGDs and KI interviews selected from villages not included in the household individual interviews were separated into different groups according to gender to encourage free expression of their view about the male involvement in IYCF. The interviews were tape recorded and notes taken.

The semistructured questionnaires and interview guides were pretested from a selected village of the neighboring Bushenyi district, Salter weight scales were adjusted back to a zero initial reading on taking weight of each child.

*Inclusion and Exclusion Criteria*. The included households had a child aged 6–59 months and a male partner/caretaker interviewed, and all participants had consented. Children who presented oedema as a clinical sign of malnutrition and WHZ above +3 SD were not included in the WHZ analysis.

*Study Variables*. Primary outcome: child nutritional status (stunting, wasting, and underweight, measured by height-for-age, weight-for-height, and weight-for-age indices of −2 SD or more, resp.), and presence of oedema. The primary independent factor, male involvement in infant and young child nutrition, was defined using 14 main explanatory variables presented in five dimensions. These were adopted from the brief description of male involvement in IYCF policy guidelines of Uganda and the different male involvement activities from literature review on engaging grandmothers and men in IYCF, a project that was implemented in Kenya [13, 14].

(1) Decision-making in child nutrition was as follows: final decision on exclusive breastfeeding, final decision on time to start complementary feeding, final decision on what food for start of complementary feeding, and final decision on order of serving food during meal times. (2) Providing physical support to the lactating mother was as follows: feeding the child during meal times, assisting mother with household chores, assisting mother with farming activities, accompanying mother for child health clinics, and allowing other family members/relatives to support the mother after delivery. (3) Providing psychological support to the lactating mother was as follows: providing appropriate information on breastfeeding to the mother. (4) Providing financial support was as follows: buying food for the child, buying food for the lactating mother, and transporting to the child health clinics. (5) Promoting optimal child feeding practices were as follows: providing information about young child feeding.

### 2.1. Statistical Analysis

Coded variables were manually entered using Excel 3.1 version and then transferred to STATA 11 for analysis.* t*-tests for continuous variables and chi-square tests for the categorical variable were done at univariate analysis. Height-for-age, weight-for-age, and weight-for-height *z*-scores were calculated using WHO Anthro version 3.2.2. Children who were considered as stunted, wasted, and underweight had a *Z* score of <−2 standard deviation. The main explanatory variable, male involvement, was assigned binary responses, (“1” (yes) representing the fact that male partner/caretaker plays a role and “0” (no) representing the fact that male partner/caretaker does not play a role). Level of male involvement was measured using individual male involvement explanatory variables and, furthermore, using an ad hoc male involvement index with all variables equally scored with 1 (yes) and 0 (no). Overall score was 14, where male respondent scored 1 in all variables considered. Scores 0–7 equal low male involvement and scores of 8–14 equal high level of male involvement. Logistic regression models were run using backward stepwise variable selection method to explore the degree to which multiple variables were associated with child nutritional status. Probability values (*p* values) were calculated at the 0.05 level of significance. The FGDs and in depth interviews were conducted in the local language (Runyankore-Rukiga) and the sessions lasted one to two hours. The data was transcribed, coded, and summarized into themes.

### 2.2. Ethical Consideration

This study was approved by the Research Ethics Committee of Mbarara University of Science and Technology. Informed consent, and assent for the children under 18 years of age, was sought from the adult study participants after clearly explaining the objective of the study, the procedures to go through to obtain data, assessing the nutritional status of the children, and the time required.

### 2.3. Study Limitations

This study did not use a standard tool to measure male involvement in child feeding and this may differ due to the sociocultural, economic, physical, biological, and behavioral contexts of the study area. Therefore the findings may not be generalized to other settings. Furthermore, since only fathers/male caretakers were interviewed, they may have given information to portray perceived better feeding in children resulting into social desirability bias. Conducting key informant and focus group discussion were techniques used to triangulate the participants' responses and the variables that were adopted for male involvement in infant and young child feeding. Also, due to logistical constraints, water sources were not verified for categorization into the conventional safe and unsafe water sources of UNICEF.

## 3. Results

A total of 415 households were eligible and 346 male respondents consented with male respondent and a child under 5 years pairs.

### 3.1. Baseline Characteristics of Participants

Table 1(a) shows the characteristics of the participants in the study.

The median age of the children was 29 months (IQR: 20.1–41.1), and 65% aged 24 to 59 months, with an equal distribution among the genders. The percentage (22%) of under-5 children who had suffered from malaria in the previous 14 days was greater compared to those who suffered pneumonia (4.1%) and diarrhea (14.2%). Thirty percent of the children were stunted, 14.8% underweight, and 7.5% wasted. The mean age of the male respondents was 40.58 years (SD: 13.2), 41% aged 36 to 50 years.

Table 1(b) shows the male and household baseline characteristics of the study participants. Over 50% of male respondents did business and only 7% had professional occupation as source of income. Over 60% of the households had food stored at the time the study. The major source of drinking water for most households, 204/346 (59%), was water from open well, and 79% of the households had a hand washing facility although only 9% reported their children usually washed hands after using the latrine.

### 3.2. Level of Male Involvement and Infant and Young Child Feeding

Table 2 shows the level of male involvement in infant and young child feeding activities. The level of male involvement was assessed using fourteen variables, assigned to five domains. Male respondents reported providing physical support with over 80% participating in farming and 94% providing money to buy food. This was further illustrated by both female and male participants during the FGDs

“I think as a man I have to take care of my family. The money I make does many things and buying  food is one of them. Sometimes the children are sick or my wife has to go to hospital for immunization, so I give her some money to use.” (Male FDG, Mwijo village)

“Men who make sure that there is enough maize flour to make the children's porridge to me are responsible. Many women will agree, that a man should make sure his family members do not sleep without eating anything or that his children are not the ones that get ‘mutuku' all the time.” (Female and Male FDG, Nyakabirizi 2)

Only 9% of male respondents reported accompanying mother to child health clinics for growth monitoring, although 47% provide money for the transport to the child health clinics. Male respondents' knowledge about breastfeeding and complementary feeding was limited as less than 40% of male respondents could correctly mention at least one message they give to mothers, about breastfeeding and complementary feeding

As illustrated, consider the following.

“For me, I don't know much of what special food a young child like my son should eat and I have no problem if he eats with other older children. My wife also can decide what they will eat. But I know that if the child is breastfeeding then no crying a lot and they don't get sick many times, and a woman who is breastfeeding should eat many times to breastfeed well.” (KI, Kasaana)

Overall, the level of male involvement was high among 65.5% of the respondents and 34.5% reported low involvement.

Table 3(a) shows the relationship between various male involvement activities and the child's nutritional status.

Providing money for transport to child health clinics was significantly associated (*p* < 0.05) with good nutritional status (odds ratio (OR) = 0.52; 95% confidence interval (CI): 0.34–0.81). Also low male involvement score was associated with undernutrition (odds ratio (OR) = 1.9; 95% confidence interval (CI): 1.13–3.12).

Fathers/male caretakers whose main occupation was business as source of income were more likely to have children with good nutritional status than those not doing business as a source of income (odds ratio (OR) = 0.6; 95% confidence interval: 0.42–0.99).

Table 3(b) shows a bivariate analysis of other factors associated with child nutritional status.

Using borehole as the main source of drinking water was protective of undernutrition compared to not using water from borehole (odds ratio (OR) = 0.5; confidence interval (CI): 0.28–0.96). However households whose main source of drinking water was a spring were 2 times more likely to have children with undernutrition compared to those not using water from spring (odds ratio (OR) = 2.0; 95% confidence interval: 1.13–3.80). Having a functional common place for hand washing protected the children from poor nutritional status compared to not having a common place to wash hands (odds ratio (OR) = 0.5; 95% confidence interval (CI): 0.32–0.90) in bivariate analysis.

Table 4 shows a multivariate analysis of the factors that were statistically significant in the bivariate analysis of factors associated with child nutrition.

The factors that were significant in the unadjusted (bivariate) analysis were fitted into a multivariable logistic model with nutritional status as the outcome. Providing money for transport to child health clinics was associated with good nutritional status (adjusted odds ratio (aOR) = 0.5; 95% confidence interval (CI): 0.32–0.79, *p* value 0.003).

Also male respondents, who had a low level of involvement in infant and young child feeding, were more likely to have children with undernutrition (adjusted odds ratio (aOR) = 1.8; 95% confidence interval (CI): 1.09–3.03, *p* value 0.022).

Finally using the spring as source of drinking water increased the risk of undernutrition 2 times than not using water from this water source (adjusted odds ratio (aOR) = 2.1; 95% confidence interval (CI): 1.1–3.9, *p* value 0.032).

## 4. Discussion

In this study the level of stunting was high compared to being underweight and wasted and was slightly lower than the level of stunting in the whole Southwestern region. This agrees with a study by FANTA II [15] which reported 3 in 10 children as stunted in this region despite being the food basket of Uganda. More children in this study area were also wasted compared to those reported in the Southwestern region in UDHS report [16]. It should be noted that the nutritional outcomes of young children result from a combination of different factors which interact at different levels, and, therefore, availability of food does not necessarily translate into good nutritional status for children under five years of age [17].

The percentage of under-five children who were exclusively breastfed in this study was higher than that reported for Uganda; however early introduction of other feeds (<6 months) was 4 times more. Replacement feeding was rare, 3 times lower than that reported for Uganda [16]. Interestingly, length of exclusive breastfeeding, mixed feeding, and replacement feeding, was not significantly associated with the nutritional status of the under-fives in this study area; this is contrary to a study reported in Eastern Uganda [18] where infant feeding mode/option was a proximate factor significantly associated with stunting among under-five children.

### 4.1. Level of Male Involvement in Infant and Young Child Feeding

The level of male involvement was high in two domains of provision of physical support and financial support and this is because culturally men are considered as providers and control resources in most households. Therefore their participation in child feeding will be affected by their roles in the household. These results agree with the study done in Kenya in [14], which describes the key role of men in child feeding as provision of food and resources to provide food for the family.

The activity that the males least participated in was decision-making in infant and young child feeding, In particular they did not participate in decision making on when to exclusively breastfeed, time to start complementary foods, type of food to start complementary feeding, and order of serving food during meal times. This finding can be attributed to the cultural norm that the responsibility of child feeding is the mother's and young children spend most of the time with their mothers. This study also found out that some men mention that working away from their homes makes them less available to participate in child feeding activities. Other studies done in Ethiopia to increase male participation in child feeding [19] and an assessment of male's participation in infant and young child feeding in Kenya by Thuita et al. [14] agree that men participate less in decision-making for child feeding. Also, few male respondents reported not accompanying mothers to child health clinics and more providing of money for transport to the child health clinics can be explained by the fact that men's attendance of maternal and child health visits is still low in Uganda. Tweheyo et al. [20] report that a low number of men accompany their wives to seek maternal and child health services in Kenya, Tanzania, and Northern Uganda.

More than half of male respondents interviewed did not know or give any appropriate message about young child feeding and breast feeding to pass on to the mothers. This may be explained by the low number of male respondents who accompany mothers to the child health clinics. Attending child health clinics together gives an opportunity for both the mother and father to access appropriate messages about child feeding from trained health workers. Also male focus group discussions expressed the desire to know more about feeding of their children. In agreement with this finding, other studies in Nepal, UK, and Uganda which are related to maternal health issues have found that limited knowledge of male partners about maternal health issues has been a significant barrier to attending skilled antenatal care, where health talks about appropriate infant and young child feeding are usually given [15, 20].

### 4.2. Relationship between Level of Male Involvement and Nutritional Status of Under-Five Children

From all the 14 activities that were considered for the study, male's involvement by providing mothers with money for transport to child health clinics was significantly associated with good nutritional status of the under-fives. The implication here is that attending child health clinics gives mothers the opportunity to get appropriate information on infant and young child feeding during health/nutrition education and counseling sessions. Mothers who attend child health clinics for growth promotion and monitoring are more likely to practice optimal infant feeding practices, which will result in good nutritional status of their children [21]. On scoring the level of male involvement, it showed that as the number of activities done by male respondents increased, the nutrition status of the children under five also improved. And although providing money for transport to child health clinics was significant, the results show that if men participate in 8 and more activities then it improves their children's nutritional status. Although other male involvement activities were not significant in this study, it should be noted that, to achieve optimal infant and young child feeding, mothers require the social support of their male counterparts since it has been noted that they are one of the key influences of child feeding. Physical, financial, and psychosocial support offered by fathers will eventually affect infant and young child feeding practices [22].

Other factors that were significantly associated with good nutritional status of the under-5 children in this study were presence of a functional hand washing facility in a household and using water from the borehole [6, 8, 23].

Also it was noted that households using water from a particular spring predisposed the children to undernutrition, and this is a finding that requires further investigation. Having a functional hand washing facility and safe water for consumption improves hygiene and sanitation hence reducing exposure to contamination which increases risk of diarrheal diseases, which is an infection that causes undernutrition in Uganda [6, 8, 23]. Interestingly age, gender, other household factors, and father's/male caretaker's education were not significantly associated with the nutritional status of the under-five children in this study area, contrary to other studies reported in Western and Northern Uganda and Zambia [24–27].

## 5. Conclusion

Six in ten male respondents participated in 8 and more activities and were more likely to have under-five children with good nutritional status than those involved in less than 8 activities. The level of male involvement in IYCF was high in the provision of physical and financial support; however a lower percentage of men are involved in decision-making for IYCF, providing information on child feeding and accompanying mothers to child health clinics. Providing transport for child health clinics positively associates with the child's nutritional status, since attending these clinics will provide an opportunity to acquire appropriate knowledge on IYCF for better feeding habits and practices among children under five years.

The choice of the feeding mode/option for the child appears not to affect the nutritional status of the children in this study.

## Figures and Tables

**Table:** (a) Baseline characteristics of the children

Characteristics	*n* (%)
Median age (IQR), months	28.7 IQR: 20.1–41.1
Gender, *n* = 346	
Male	176 (51.6)
Female	168 (48.4)
Length of exclusive breastfeeding (months), *n* = 346	
0–3	31 (8.9)
4–6	252 (72.8)
Don't know	63 (18.2)
Age at complementary feeding (months) *n* = 346	
<6	60 (17.3)
6	176 (50.9)
7–9	54 (15.6)
Do not know	56 (16.2)
Pneumonia, *n* = 346	14 (4.1)
Malaria, *n* = 346	78 (22.5)
Diarrhea, *n* = 346	49 (14.2)
Underweight, *n* = 346	51 (14.8)
Wasted, ^*∗*^*n* = 345	26 (7.5)
Stunted, *n* = 346	107 (30.9)

^*∗*^One child had bilateral edema, not included in analysis. IQR: Interquartile Range.

**Table:** (b) Male and Household baseline characteristics

Characteristics	*n* (%)
Mean age (years)	40.58 (SD: 13.21)
Age in years, ^*∗*^*n* = 344	
≤25	20 (5.8)
26–35	123 (35.8)
36–50	142 (41.3)
>50	59 (17.2)
Occupation, ^*∗∗*^*n* = 346	
Business	186 (53.8)
Manual labor	129 (37.3)
Professional	25 (7.2)
Peasant farmer	131 (37.9)
Level of education, *n* = 346	
No formal education	37 (10.7)
Primary	226 (65.3)
Secondary	47 (13.6)
Tertiary	36 (10.4)
Marital status, *n* = 346	
Single	7 (2.0)
Married	335 (96.8)
Widower	1 (0.3)
Separated	3 (0.9)
Household characteristics	
Food security, ^*∗∗∗*^*n* = 233	
Food secure	46 (19.7)
Mildly food insecure	10 (4.3)
Moderately food insecure	74 (31.8)
Severely food insecure	103 (44.2)
Water source, *n* = 346	
Piped water, dwelling	12 (3.5)
Piped water, communal tap	30 (8.7)
Borehole	60 (17.3)
Open well	204 (59)
Surface water, stream	11 (3.2)
Spring	50 (14.5)
Rain water	74 (21.4)
Hand washing facility, *n* = 346	273 (78.9)

^*∗*^One child presented oedema, WHZ not included. ^*∗∗*^Two male respondents did not know their age in years. ^*∗∗∗*^Male respondent has more than one occupation. ^*∗∗∗∗*^113 households had missing data on household food security.

**Table 2:** Level of involvement of 346 males in infant and young child feeding activities.

Activity	Respondent's responses	
	Yes	No
*Decision making in infant and young child feeding *		
Final decision on exclusive breastfeeding	67 (19.4)	279 (80.6)
Final decision on time to start complementary foods	79 (22.8)	267 (77.2)
Type of food for start of complementary feeding	79 (22.8)	267 (77.2)
Order of serving food	68 (19.7)	278 (80.4)
*Providing physical support*		
Participate in child feeding	178 (51.5)	168 (48.6)
Assist in household chores	255 (73.7)	91 (26.3)
Allows help after delivery	258 (77.5)	75 (22.5)
Assist in farming	287 (82.9)	59 (17.01)
Accompanying mother to child health clinics	34 (9.8)	312 (90.2)
*Support and guidance for Breastfeeding*		
Provide appropriate information about breastfeeding^**∗**^	124 (41.5)	175 (58.5)
*Financial support*		
To buy food for the child	324 (93.6)	22 (6.4)
To buy food for lactating mother^**∗∗**^	218 (72.9)	81 (27.1)
Transport money to child health clinic	163 (47.1)	183 (52.9)
*Promoting optimal child feeding practices*		
Provide appropriate information about young child feeding	154 (44.5)	192 (55.5)

^*∗*^Male respondents not living with child during breastfeeding period and ^*∗∗*^male respondents not living with the mother during lactation period.

**Table:** (a) Relationship between various male involvement activities and the child nutritional status

Activity (yes)	COR (95% CI)	*p* value
Final decision on exclusive breastfeeding	0.56 (0.32–1.01)	0.049
Final decision on time to start complementary foods	0.72 (0.43–1.23)	0.223
Type of food for start of complementary feeding	0.69 (0.42–1.15)	0.158
Order of serving food	1.05 (0.61–1.81)	0.854
Participate in child feeding	0.87 (0.56–1.34)	0.517
Assist in household chores	0.70 (0.43–1.14)	0.151
Allows help after delivery	0.81 (0.48–1.38)	0.443
Assist in farming	0.77 (0.43–1.35)	0.358
Accompanying mother to child health clinics	1.12 (0.55–2.30)	0.758
Provide appropriate information about breastfeeding	1.08 (0.67–1.73)	0.752
To buy food for the child	0.61 (0.26–1.45)	0.268
To buy food for lactating mother	0.92 (0.55–1.59)	0.765
Transport money to child health clinic	0.52 (0.34–0.81)	0.004^*∗*^
Provide appropriate information about young child feeding	0.76 (0.49–1.17)	0.21

^*∗*^ *p* < 0.05: statistically significant, CI: confidence interval at 95%, *p* value: probability value at 0.005, and COR: Crude Odds Ratio.

**Table:** (b) Bivariate analysis of other factors associated with child nutritional status

Variable	COR (95% CI)	*p* value
*Length of exclusive breast feeding*		
0–3	1	
4–6	0.93 (0.43–1.99)	
Do not know	1.35 (0.56–3.24)	0.418
*Mixed feeding*		
No	1	
Yes	0.89 (0.50–1.55)	
Do not know	1.27 (0.63–2.57)	0.694
*Replacement feeding*		
No	1	
Yes	0.89 (0.29–2.70)	
Do not know	2.39 (0.39–14.52)	0.613
*Occupation*		
Business	0.64 (0.42–0.99)	0.046^*∗*^
Manual labor	1.37 (0.88–2.13)	0.169
Professional	0.73 (0.31–1.74)	0.468
Peasant farmer	1.01 (0.65–1.58)	0.952
*Water source*		
Piped water, dwelling	1.14 (0.35–3.65)	0.832
Piped water, communal tap	0.55 (0.24–1.27)	0.146
Borehole	0.52 (0.28–0.96)	0.031^*∗*^
Open well	1.22 (0.79–1.91)	0.369
Surface water, stream	N/A	0.000
Spring	2.07 (1.13–3.79)	0.018^*∗*^
Rain water	0.95 (0.56–1.62)	0.859
*Hand washing facility*		
No	1	
Yes	0.54 (0.32–0.90)	0.019^*∗*^

^*∗*^ *p* < 0.05: statistically significant, CI: confidence interval at 92%, *p* value: probability value at 0.005, and COR: Crude Odds Ratio.

**Table 4:** Multivariable analysis of factors associated with child nutritional status.

Independent variable	COR (95% CI)	aOR (95% CI)	*p* value
Transport money to child health clinic			
No	1	1	
Yes	0.52 (0.34–0.81)	0.49 (0.32–0.79)	0.003^*∗*^
Hand washing facility			
No	1	1	
Yes	0.54 (0.32–0.90)	0.55 (0.32–0.94)	0.028^*∗*^
Spring	2.07 (1.13–3.79)	2.13 (1.14–3.96)	0.018^*∗*^

^*∗*^ *p* value < 0.05, statistically significant, LR = 19.69, *R*^2^ = 0.048, model *p* value = 0.694, Hosmer-Lemeshow chi^2^ = 1.45. COR: Crude Odds Ratio. aOR: adjusted odds ratio.
